# Promoting community reintegration using narratives and skills building for young adults with stroke: a protocol for a randomised controlled trial

**DOI:** 10.1186/s12883-020-02015-5

**Published:** 2021-01-04

**Authors:** Suzanne Hoi Shan Lo, Janita Pak Chun Chau, Kai Chow Choi, Edward Wai Ching Shum, Jonas Hon Ming Yeung, Siu Hung Li

**Affiliations:** 1 The Nethersole School of Nursing, Faculty of Medicine, Chung Chi College, The Chinese University of Hong Kong, Shatin, N.T., Hong Kong SAR, China; 2 New Territories East Cluster, Hospital Authority, Hong Kong SAR, China; 3 Department of Medicine, Alice Ho Miu Ling Nethersole Hospital, Hospital Authority, Hong Kong SAR, China; 4 Department of Medicine, North District Hospital, Hospital Authority, Hong Kong SAR, China

**Keywords:** Stroke, Narration, Social participation, Self-management, Randomised controlled trial

## Abstract

**Background:**

Stroke in adults aged between 18 and 64 years old is increasing significantly worldwide. Studies have reported that this group of young stroke survivors encounters enormous difficulties reintegrating into their social roles. Individualised discussions with healthcare professionals and learning from other survivors are imperative for them to reconstruct their identities after stroke. There is also great demand for community support during their chronic stage of recovery to help them rebuild life skills to promote reintegration.

**Methods/design:**

This is a randomised controlled trial to investigate the effects of a 24-week Narrative and Skills-building Intervention (NSI) on young stroke survivors’ community reintegration and psychosocial outcomes. A total of 208 adults aged 18–64 years old with a first-ever or recurrent ischaemic or haemorrhagic stroke and have been discharged home will be recruited and randomly assigned to receive usual care or usual care with NSI. The NSI is grounded in Narrative Theory and Bandura’s principles of Self-efficacy and Outcome Expectation, consisting of successive eight individual sessions over six months delivered by a trained facilitator (a registered nurse). Participants will be facilitated to narrate their survival experiences and rebuild core life skills. Videos of peer young stroke survivors’ experiences of recovery will be provided. Outcomes including community reintegration, depressive symptoms, health-related quality of life, self-efficacy, outcome expectation and satisfaction with performance of self-management behaviours will be measured before (T0) and immediately after NSI (T1), then six (T2) and 12 months after NSI (T3). Generalised estimating equations models will be used to compare the differential changes in outcomes across time between the two groups. Focus group interviews will be conducted with the facilitator at T1 and with the participants in the intervention group at T1 and T3.

**Discussion:**

This study will evaluate the short and long-term effects of a theory-based NSI on young stroke survivors’ community reintegration and establish a new model of community reintegration after stroke to inform future research. The results will also provide valuable evidence to develop clinical guidelines for young stroke survivors’ community reintegration.

**Trial registration:**

ClinicalTrials.gov identifier: NCT04560140, registered on 23 September, 2020.

**Supplementary Information:**

The online version contains supplementary material available at 10.1186/s12883-020-02015-5.

## Background

The global prevalence of stroke in adults aged 20–64 years old doubled from 1990 to 2013 [1]. International guidelines recommend promoting community reintegration as a rehabilitation priority for stroke survivors [2, 3]. Community reintegration refers to one’s ability to resume or adapt one’s roles and responsibilities and to actively participate in social activities after a disabling illness [4]. Studies have reported that this group of young adults with stroke encounters substantially greater difficulties reintegrating into their social roles compared to older adults. Common challenges include maintaining family relationship and a social network, resuming leisure activities, or returning to work [5, 6]. Age-appropriate interventions to facilitate young stroke survivors’ acceptance of their functional limitation and development of life skills to reconstruct their roles for better community reintegration are warranted.

Previous studies have consistently reported the complex and chronic nature of young stroke survivors’ community reintegration [5–8]. A review of the literature on survivors aged 18–65 years old found that the abrupt onset of stroke was often stigmatised as a disease of old age, and that their ability to perform self-care activities was consequentially compromised, which significantly disrupted their sense of self, role and relationship. They had a loss of control and an increased sense of uncertainty in their life after stroke [5]. A qualitative study of 17 community-dwelling survivors aged 23–55 years old reported that they might develop an adapted sense of self several years after the stroke event. However, it has been suggested that these survivors need guidance and support to reflect on their experiences, re-appraise their strengths and re-adjust their personal values to re-construct their identities and roles [6]. A qualitative study of 22 stroke survivors who were aged 20–61 years old and about six months to nine years after stroke found that their struggles of inability to fulfil their role expectations or maintain intimate relationships resulted in emotional shortcomings such as feelings of isolation or helplessness. The results highlighted the need for providing survivors with opportunities to narrate their experiences outside their homes and offering support in the later phase of recovery to prevent psychosocial problems [7]. Another study of 12 women aged 18–50 years old has also highlighted the young stroke survivors’ unmet needs, including a lack of access to age-appropriate information, inadequate professional support regarding life skills training, limited dialogue with healthcare professionals and underuse of peer learning to support the continual reintegration process [8].

Personal or illness experience narratives have been examined in previous studies to help stroke survivors re-establish their identity and roles through meaning-making, bridging between the present situation and the ideal future life, and exploring opportunities for change [9–11]. A feasibility study of 17 survivors aged 33–89 years old reported that those receiving a nurse-led intervention using narratives appreciated the opportunities to share and re-create their stroke experience with a healthcare professional. Following Narrative Theory, the participants were supported to reflect on their experience through this act of narrating. The narrative facilitated their reflection and self-understanding. It stimulated them to envision for future changes and make adjustment in their lives after stroke [10]. A one-group study of 27 survivors aged 44–73 years old with post-stroke aphasia found that a biographic-narrative intervention was associated with potential benefits in health-related quality of life (HRQoL) and mood at three months post-intervention. Participants were asked to share their daily life experiences after stroke to reshape their identity. They were also facilitated to learn from peers and increase social contact via discussing health or leisure time issues during group sessions [11].

Skills-building is vital to improve stroke survivors’ ability to plan, solve problems and set personal goals for better community reintegration [12]. Three experimental studies examined the effects of self-management interventions aimed at promoting participation in daily life after stroke. The interventions, led by an occupational therapist, focused on home modifications and developing survivors’ self-management skills, including problem solving, decision making, action planning, resource utilisation and partnership with care providers [13–15]. Among the studies, one randomised controlled trial (RCT) of 185 adults with mild to moderate stroke found significant improvements in health-related and participation self-efficacy in daily life activities immediately after completing the 12-session group and community-based intervention [13]. A follow-up quasi-experimental study of 17 survivors found significant improvement in community reintegration upon completion of a 6-session intervention enriched with components for problem solving and goal setting [14]. A randomised pilot study of 15 survivors also reported a clinically meaningful change in social participation at six months after receiving a participation-focused individual-based programme that included one pre-discharge and five post-discharge home visits [15]. Several systematic reviews have suggested that problem-solving skills are likely to facilitate self-management after stroke [12, 16, 17]. Contrastingly, an RCT of 62 stroke survivors reported significant improvements in depressive symptoms and anxiety among those who received a solution-focused therapy compared with the control group [18]. Two qualitative studies of 12 stroke survivors also suggested doing everyday activities at home developed skills in managing role changes through mastery experiences and negotiation with spouses [19, 20].

There have been limited studies that examined interventions for promoting community reintegration after stroke, and young stroke survivors are often under-represented in these studies. Moreover, grounding the design, development and evaluation of such a complex intervention in a theoretical framework is important to explain the mechanisms underlying the changes in outcomes [21]. More investigations on the best theoretical frameworks that consider a person’s interpretation of experiences and self-efficacy relating to behavioural change are needed. There are an increasing number of studies that have used narratives as an approach to help stroke survivors to understand their experience of stroke and have showed positive results for psychological well-being. More evidence on the effects of using narratives in promoting the community reintegration of young stroke survivors would be worthwhile. Young stroke survivors need to develop life skills to address their internal struggles and the challenges they encounter during the return to their family and social roles. There has been no consensus, however, on the core skills for promoting community reintegration in young stroke survivors.

This study aims to investigate the effects of a 24-week Narrative and Skills-building Intervention (NSI) on community reintegration, self-efficacy, outcome expectation, satisfaction with performance of self-management behaviours and psychosocial outcomes among young stroke survivors. We hypothesise that the participants in the intervention group, compared with the control group, will demonstrate all of the following at immediately, 6 months and 12 months post-intervention: a) Significant increase in community reintegration with respect to baseline; b) Significant improvements in depressive symptoms, HRQoL and satisfaction with performance of self-management behaviours with respect to baseline, and c) Significant increases in self-efficacy and outcome expectation of performing self-management behaviours with respect to baseline.

## Methods/design

### Study design

**Fig. 1:**
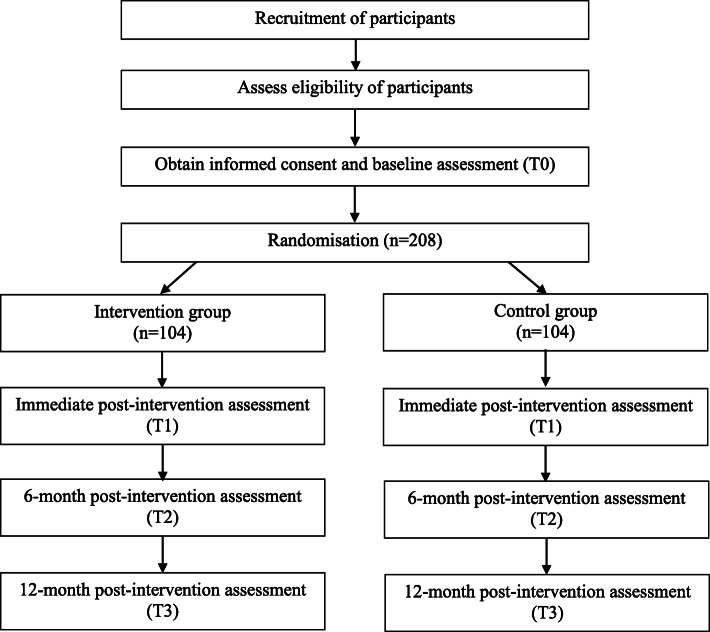
Study flow diagram

A multi-centred, assessor-blinded, two-arm RCT will be conducted. The outcomes will be measured at baseline (T0), immediately post-intervention (T1), and 6 months (T2) and 12 months post-intervention (T3) (Fig. 1).

### Setting

Participants will be recruited from three acute public hospitals, and community-based organisations serving people with chronic conditions including stroke in Hong Kong. The three hospitals follow the same stroke care pathway endorsed by the Hong Kong Hospital Authority. All sessions of the NSI and all of the assessments will be conducted in the participant’s homes or a community centre.

### Participants

Individuals will be recruited if they: (1) are 18–64 years of age, (2) have a clinical diagnosis of first-ever or recurrent ischaemic or haemorrhagic stroke, (3) are living at home after discharge from hospital, (4) have a Montreal Cognitive Assessment (MoCA) score > 2nd percentile, (5) have a modified Rankin Scale (mRS) score ≥ 3 (moderate disability), (6) can communicate in Cantonese, and (7) are able to attend all NSI sessions.

Individuals will be excluded if they 1) have been diagnosed with transient ischaemic attack, subdural or epidural haemorrhage [22], 2) have experienced cerebrovascular events due to tumours or head trauma, 3) have been diagnosed with a mental condition such as depression, schizophrenia, bipolar or personality disorder, 4) demonstrate incomprehensible speech or difficulty in comprehending conversations, or 5) have received a self-management programme in the past.

### Sample size estimation

The sample size is estimated based on the outcomes of community reintegration, self-efficacy and outcome expectation. In our previous interventional study [23], the effect sizes of a stroke self-management programme on community reintegration, self-efficacy and outcome expectation of performing self-management behaviours were 0.44, 0.55 and 0.53, respectively. Using the power analysis software PASS 16 (NCSS, LLC. Kaysville, Utah, USA), a sample size of 83 participants per study arm is estimated to give this two-arm RCT 80% power at a two-sided 5% level of significance to detect an effect size as small as 0.44 on the primary outcome between the control and intervention groups at a post-intervention time point. Further allowing for a potential attrition rate of up to 20%, 208 participants, with 104 per group, will be recruited.

### Randomisation and blinding

The participants will be randomly allocated to either the intervention (I) or the control (C) group at 1:1 ratio after they have provided consent and baseline data. Block randomisation in blocks of ten, except the last block, will be used. An independent statistician will generate a random sequence of grouping identifiers (I or C) using a random list generator (www.randomization.com). According to the sequence, a grouping identifier will be placed into an opaque, identical, sealed and sequentially numbered envelope for concealed allocation. All participants will be pooled into the same enrolment list regardless of their recruitment venues. A research assistant will open the envelopes sequentially according to the participants’ time of enrolment and record the grouping. Another research assistant who will conduct the baseline and follow-up assessments and data entry will have no knowledge of the participants’ group assignments. Blinding is not possible for the participants and the facilitator who will deliver the NSI, due to the nature of the intervention. The research assistant, who will conduct qualitative evaluation with the facilitator and participants’ feedback on the NSI, will know the group allocations.

### Intervention

The NSI aims to promote community reintegration of young stroke survivors and is designed on the basis of Narrative Theory [24] and Bandura’s principles of Self-efficacy and Outcome Expectation [25]. The NSI will consist of eight face-to-face sessions (1.5–2 h each) to be delivered over 24 weeks by a registered nurse (facilitator). The NSI will blend narratives and skills-building in each session to support participants’ reintegration into their post-stroke social roles. The narrative part will elicit the participants’ narratives of their experiences of engaging in everyday social activities including social, leisure and work activities, and will be used to identify the participants’ strengths, co-construct meanings of their experiences, re-structure self-identity and social roles, envision the future and explore opportunities for change. The skills-building part will develop the participants’ abilities to apply the life skills learned in order to maintain health and sustain their engagement in social activities. Three types of life skills will be included: self-management (goal setting, action planning, decision making, communication and utilisation of resources), outcome-expectation enhancing (reflection, solution focused) and self-efficacy enhancing (mastery, modelling, persuasion and reinterpretation of physical and emotional arousal) [12–20, 23].

Each session will have an assigned themed topic to articulate the narratives and skills-building. Each session is designed with a theme-related activity to arouse the participants’ interest in narrating their experiences and discussing the assigned themed topic. Strategies informed by the theoretical framework and tailored to young stroke survivors’ health needs will be adopted. The first three sessions will be foundational and conducted at the participants’ home bi-weekly to engage the participants in applying the skills learned to perform everyday household and social activities. The participants’ family members or caregivers will be invited to join these sessions. The remaining five sessions will be reintegration-enhancement sessions conducted in a private room at a community centre once every three weeks. These sessions will encourage the participants to address their struggles while reconstructing their self-identities and social roles. Each participant will be provided with a workbook with age-appropriate quick references for their narration and skills-building. Each participant will be given an online account to record their goals, action plans and daily accomplishments, and to retrieve 15 videos of other young stroke survivors sharing their experiences of reintegrating into social roles.

An expert panel consisting of two academics, one physician, one nurse consultant, two ward managers and one social worker will review the contents of the NSI. Their feedback will be incorporated in further revisions if deemed necessary. The revised NSI will be piloted with 10 potential young stroke survivors to assess their satisfaction and acceptability. Revisions will be made with consideration of the survivors’ feedback.

### Usual care

The usual care will include the usual stroke care services available to the participants during the study period. It will include, but not be limited to, medical consultations or rehabilitation services offered by the hospital or health organisations.

### Quantitative outcome measures

#### Primary outcome

##### Community reintegration

The Chinese version of the Reintegration to Normal Living Index (RNIL-C) will be used to assess the participants’ level of community reintegration [26, 27]. It consists of 11 items in eight domains: mobility, self-care, daily work and school activity, recreational and social activities, family roles, personal relationships, presentation of self to others and general coping skills. Participants will be asked to rate the extent to which each item describes their situation on a scale from 1 – ‘a small extent’ to 5 – ‘a great extent’. The total score is calculated by summation and normalised to give 100 with a total score range of 25 to 100. A higher score indicates better community reintegration. The RNIL-C has high internal consistency (Cronbach’s alpha = 0.92) and good convergent validity [27].

#### Secondary outcomes

##### Depressive symptoms

The Chinese version of the 15-item Geriatric Depression Scale will be used to measure participants’ depressive symptoms [28, 29]. Studies support its utility in younger adults (> 18 years old), with good diagnostic sensitivity and specificity [30, 31]. Each item represents symptoms of depression and describes a participant’s condition in the preceding week; the participants answer each item with either ‘yes’ or ‘no’. All items are summed (total score 0–15). A score of six or greater is a cutoff for depression. The scale has a Cronbach’s alpha of 0.78 [29].

##### HRQoL

Participants’ HRQoL will be measured by the Chinese version of the Stroke-Specific Quality of Life Scale [32, 33], which has 47 items with 11 domains ranging from physical to psychosocial and participation. The items are about the health conditions of the participants and how much difficulty the participants have when doing everyday self-care tasks. The items are scored from 1 – ‘strongly disagree/cannot do it’ to 5 – ‘strongly agree/no trouble’. Total score is yielded by summing all item scores (range 47–235): the higher the score, the higher the HRQoL. It has acceptable internal consistency (Cronbach’s alpha: 0.63–0.93) and convergent validity [33].

##### Self-management behaviours

The 11-item Chinese version of the Stroke Self-management Behaviours Performance Scale will be adopted to assess participants’ satisfaction with the performance of self-management behaviours. Each item is scored using a range from 0 – ‘very dissatisfied’ to 10 – ‘very satisfied’. Taking the sum of all item scores yields one total score (range 0–110), and the higher the score, the higher the satisfaction. This scale has a Cronbach’s alpha of 0.93 [23].

##### Self-efficacy

The Chinese version of the Stroke Self-Efficacy Questionnaire will be used to measure self-efficacy [34, 35]. It has 13 items, each is scored using a scale from 0 – ‘no confidence’ to 10 – ‘very confident’. The items are about the participants’ perceived extent of confidence in doing everyday activities and self-management tasks. A total score is yielded by summing all items (range 0–130). A higher total score represents higher self-efficacy. The scale has acceptable internal consistency (Cronbach’s alpha = 0.92) and convergent validity [35].

##### Outcome expectation

The Chinese version of the Stroke Self-management Outcome Expectation Scale will be used to measure the participants’ outcome expectation beliefs. It has 11 items, each rated using a scale from 0 – ‘strongly disagree’ to 10 – ‘strongly agree’. The score of each item indicates the participants’ confidence in the expected outcomes to occur. All item scores will be calculated by summation (total score 0–110). A higher score represents higher outcome expectations. This scale has good internal consistency (Cronbach’s alpha = 0.94) [23].

### Qualitative evaluation

Focus group interviews with participants who received the NSI will be conducted at a community centre immediately and 12 months post-intervention. A convenience sample of 56 participants (7 per group) will be invited. The participants will be asked to share their experience and satisfaction with the NSI, views on usefulness of the NSI in initiating and supporting their engagement in valued everyday activities, family and social roles, met and unmet health needs, and areas for enhancement. The facilitator who delivered the NSI will also be interviewed using a semi-structured interview guide immediately after the completion of the intervention to elicit feedback on the facilitators of and barriers to implementation of the NSI.

### Demographic and clinical information

Data will be recorded on the participants’ age, gender, educational level, marital status, occupation, current financial aids received, type of housing, living condition, past and present medical history, and assistive aids used. Scales such as the MoCA, the Barthel Activities Daily Living Index, mRS and the National Institutes of Health Stroke Scale will be administered. Information about the locations of stroke lesions will also be collected.

### Data collection

A research assistant will pay regular visits to the hospitals and community-based organisations to screen for potentially eligible participants. The research assistant will contact potential participants by phone and perform a preliminary eligibility assessment. The study aim, intervention, data collection procedure and rights to confidentiality will be explained to eligible patients. Participants’ written informed consent will be obtained before commencing data collection. All consenting participants will be scheduled for baseline (T0) and follow-up assessment (T1, T2 and T3 immediately, 6 months and 12 months post-intervention, respectively). Focus group interviews with the participants in the intervention group (at T1 and T3) and the facilitator (at T1) will be conducted. All interviews will be audio-taped.

### Analyses

Data will be summarised and presented using the appropriate descriptive statistics. The normality of the continuous variables will be assessed using skewness and kurtosis statistics and normal probability plot. The appropriate transformations will be made on the skewed variables before subjecting them to inferential analysis if needed. The homogeneity of the participants’ characteristics between the two study groups will be evaluated by independent t, chi-square or Fisher’s exact tests, as appropriate. The outcome analysis will be performed in accordance with the intention-to-treat principle. The generalised estimating equations models will be used to compare the differential changes in each of the primary and secondary outcomes across time between the two groups. All of the statistical analyses will be performed using SAS release 9.4 (SAS Institute, Cary, NC). All statistical tests involved will be two-sided, with the level of significance set at 0.05. All interview data will be transcribed verbatim. The transcripts will be coded and analysed by two independent researchers. The codes will be grouped to form major themes and sub-themes that correspond to the study aim and objectives.

### Ethical considerations

The study has been approved by the Joint Chinese University of Hong Kong-New Territories East Cluster Clinical Research Ethics Committee (Ref. 2019.010). The research team will protect the participants’ rights and safety by adhering to local laws, the Declaration of Helsinki, institutional policies, and the International Conference on Harmonisation-Good Clinical Practice. All eligible participants will be required to provide written consent.

## Discussion

The NSI will fill the service gap by addressing young stroke survivors’ ongoing psychological and skill-based needs to reintegrate into their social roles. Founding a complex intervention such as NSI on theoretical frameworks is crucial to explain the causal relationships between the intervention and the changes in outcomes. The results will enrich our knowledge of the practical application of integrating Narrative Theory and the principles of Self-efficacy and Outcome Expectation in the community reintegration of young stroke survivors. This novel RCT will test the short and long-term effects of a theory-based NSI on young stroke survivors’ community reintegration and establish a new model of community reintegration after stroke to inform future research. The results will provide valuable evidence to develop clinical guidelines for survivors’ community reintegration, which are not currently widely available. The NSI is expected to be integrated into existing stroke care services to benefit young stroke survivors in the long run.

## Supplementary Information

**Additional file 1.**

## Data Availability

Not applicable.

## Abbreviations

HRQoL: Health-related Quality of Life
MoCA: Montreal Cognitive Assessment
mRS: modified Rankin Scale
NSI: Narrative and Skills-building Intervention
RCT: Randomised Controlled Trial
RNIL-C: Chinese version of the Reintegration to Normal Living Index
