# Supplementary material for: Promoting community reintegration using narratives and skills building for young adults with stroke: a protocol for a randomised controlled trial
Source: BMC Neurol. 2021 Jan 4;21:3. doi: 10.1186/s12883-020-02015-5 (PMC7783975; doi:10.1186/s12883-020-02015-5)
Supplement: Supplementary file 1 — Additional file 1. [file 12883_2020_2015_MOESM1_ESM.pdf]

Information Sheet and Informed Consent Form for Stroke Participants

Introduction

Project title:	Promoting community reintegration using narratives and skills building for young adults with stroke: A randomised controlled trial
Principal investigator:	Professor Suzanne Hoi Shan LO
Department:	The Nethersole School of Nursing, Faculty of Medicine, The Chinese University of Hong Kong
Funding source:	Early Career Scheme, University Grants Committee
Reference No.:	24608019

Description of the study

The aim of this study is to investigate the effects of a 24-week Narrative and Skills-building Intervention on young stroke survivors' community reintegration and psychosocial outcomes.

Data collection procedure & questions to be asked

If you consent to participate in the project, you will be randomly assigned to join either an intervention group or a control group. If you are assigned to join the intervention group, you will participate in the 24-week Narrative and Skills-building Intervention which you will receive a total of eight individual sessions (about 1.5-2 hours each) over 24 weeks. The first three sessions will be conducted at your home, and the remaining five sessions will be conducted in a community centre. The programme will be delivered by a registered nurse. If you are assigned to join the control group, you will continue with your usual stroke rehabilitation services offered by the hospitals or other health facilities.

Regardless of the group that you join, you will also be invited to participate in four assessment to be held before joining the group, and at six, 12 and 18 months afterwards. A research assistant will conduct the assessment with you at a community centre or a university office using a structured questionnaire. Each assessment will take about an hour.

Examples of the questions included in the questionnaire: rating the appropriateness of items in describing your situations, such as "I move around my living quarters as I feel necessary", "Are you basically satisfied with your life?", "Did you have trouble preparing food?", "Exercise regularly"; and your level of confidence in "Get yourself comfortable in bed every night", "If I exercise regularly, I will be more physically independent to do what I want".

If you are allocated to the intervention group, you may also be invited to participate in a focus group interview conducted by a research assistant at immediately after completion of the intervention and at 12 months afterwards. The purpose of the interview is to collect your feedback on the intervention. The interview will take place at a community centre and will take about two hours. Examples of the questions that you will be asked: "Please tell me more about your experience in the intervention", "Tell me more about the things in the intervention that are most helpful to you", "What would you suggest to further enhance the intervention?". The interview will be audiotaped and the audio records will be duplicated in order to avoid losing data in the event that they will accidentally erase or damage.

You will also be invited to complete a questionnaire before the study to let us know about your demographic and clinical information, including age, gender, history of stroke, current health condition, physical mobility and occupation. It will take about ten minutes to complete the questionnaire.

Target participants
We will invite a total of 208 young stroke survivors to participate in the study.

Potential risks
We believe there are minimal risks with your participation in this study, which you should consider:

- The questions asked of participants in the intervention group during the individual sessions, recalling your health problems, may cause you some discomfort. If you become emotional, the sessions will be stopped. It is not the intention of the study to portray any emotional discomfort. You do not have to talk about any issue(s) you are uncomfortable sharing.

Potential benefits
The results of this project would provide valuable evidence on the usefulness of the intervention in promoting young stroke survivors' health.

Compensation
You will be reimbursed for your time involved in participation in the study. Cash incentive will be given to you upon completion of the study i.e., a total value of HK$20 if you have completed each data collection session or the interview regardless of the group that you are in.

Information protection
All the information collected from this study will be anonymous. Your name will not be disclosed in any documents or research publications related to this study. All the information will be used for research purposes only and will be kept strictly confidential. All data will be stored in a locked cabinet and only the research team members can gain access to the data. All information collected will be destroyed six years after completion of this study. The Joint Chinese University of Hong Kong – New Territories East Cluster Clinical Research Ethics Committee is an authorised regulatory authority to access the information of the participants enrolled in this study for purposes related to ethics application.

Voluntary participation
Your participation in this project is entirely voluntary. You have the right to refuse to participate or withdraw your consent to participate in the project at any time. Whether you choose to participate or not, it will not result in any negative consequences to your current activities in the health facilities.

Contact details
If you have any questions about the study, you are welcome to contact the Principal Investigator Professor Suzanne Hoi Shan LO, Assistant Professor of the Nethersole School of Nursing, Faculty of Medicine, The Chinese University of Hong Kong at phone 3943 4485 or email: suzannelo@cuhk.edu.hk. If you have questions related to your rights as a research participant, you may contact the Joint Chinese University of Hong Kong – New Territories East Cluster Clinical Research Ethics Committee at 3505 3935 or email: crec@cuhk.edu.hk.

Statement of Consent - Informed Consent Form for participating in the study (for participants)

Project title: Promoting community reintegration using narratives and skills building for young adults with stroke: A randomised controlled trial

I have read the above information and have received answers to any questions I asked. I understood the nature of this study and agree that the information collected will be kept by the researcher for at least six years beyond the end of the study.

By signing below I indicate my consent to:
- ☐ Take part in the study.
- ☐ Audio-recording during the focus group interview.

Signature of Participant:	____________________ (Printed Name:)	Date:	________
Signature of Person Obtaining Consent:	____________________ (Printed Name:)	Date:	________
Witness:	____________________ (Printed Name:)	Date:	________

Thank you for helping with this research project.
Please keep this sheet for your information.
